# Using self-affirmation to increase intellectual humility in debate

**DOI:** 10.1098/rsos.220958

**Published:** 2023-02-01

**Authors:** Paul H. P. Hanel, Deborah Roy, Samuel Taylor, Michael Franjieh, Chris Heffer, Alessandra Tanesini, Gregory R. Maio

**Affiliations:** ^1^ Department of Psychology, University of Essex, Colchester Campus, CO4 3SQ Colchester, UK; ^2^ Department of Psychology, University of Bath, Bath, UK; ^3^ Faculty of Arts and Social Sciences, University of Surrey, Guilford, UK; ^4^ School of English, Communication and Philosophy, Cardiff University, Cardiff, UK

**Keywords:** intellectual humility, debate, value affirmation, emotions

## Abstract

Intellectual humility, which entails openness to other views and a willingness to listen and engage with them, is crucial for facilitating civil dialogue and progress in debate between opposing sides. In the present research, we tested whether intellectual humility can be reliably detected in discourse and experimentally increased by a prior self-affirmation task. Three hundred and three participants took part in 116 audio- and video-recorded group discussions. Blind to condition, linguists coded participants' discourse to create an intellectual humility score. As expected, the self-affirmation task increased the coded intellectual humility, as well as participants’ self-rated prosocial affect (e.g. empathy). Unexpectedly, the effect on prosocial affect did not mediate the link between experimental condition and intellectual humility in debate. Self-reported intellectual humility and other personality variables were uncorrelated with expert-coded intellectual humility. Implications of these findings for understanding the social psychological mechanisms underpinning intellectual humility are considered.

## 1. Using self-affirmation to increase intellectual humility in debate

Regular healthy public debates signal a progressive and democratic society, but meaningful interactions and reciprocal attitude change can only occur if respective parties listen to each other in an open-minded, calm and tolerant way [1]. Unfortunately, this is not a common experience during debate, with many people experiencing stress and frustration from conversations with which they disagree [2]. Debaters often insult the competence of others, engage in ad hominem attacks, show no willingness to even consider changing their perspectives when presented with alternatives and fail to accept their own limitations. These divisive characteristics pose a risk to democratic coherence in societies, potentially exacerbating violent conflict and putting individuals materially and psychologically at risk.

An antidote to this situation, intellectual humility, is receiving increasing attention. Philosophers understand intellectual humility as the disposition to own or accept one's intellectual shortcomings out of a genuine desire for knowledge and truth [3,4]. This orientation is often contrasted with intellectually arrogant and intellectually servile behaviour, which obstruct the acquisition of knowledge and understanding [5,6]. Intellectual arrogance entails expressing an unquestioned superiority in one's attitudes, and intellectual servility entails expressing an uncritical, reflexive agreement with others (regardless of their veracity or personal views) [3].

Psychologically, intellectual humility has proven difficult to define and study. Tangney [7] suggested that it contains multiple elements simultaneously (e.g. accurate self-assessment, low self-focus, openness to new ideas) and that it is simply unamenable to direct self-report methods [8]. Nevertheless, subsequent psychological research has leaned toward viewing intellectual humility as a set of characteristics that expedite prosocial feelings and is based on prosocial values [9], and several self-report measures of intellectual humility have been developed and validated [10–12]. For example, in a series of studies, Leary *et al*. [12] found that those high in intellectual humility could detect whether a persuasive argument was weak or strong compared with those low in intellectual humility. Furthermore, self-reported intellectual humility is associated with a more accurate assessment of one's general knowledge [13] and with higher scrutiny of misinformation [14]. However, each measure of intellectual humility taps into partly unique aspects of the construct [13], which makes it difficult to identify a single gold standard approach from these measures.

The purpose of the present research was to form an *in vivo* operationalization of intellectual humility during debate, while testing an intervention to increase intellectual humility in the debating context. Past research focused on defining, operationalizing and developing instruments to measure intellectual humility. Only a few studies directly investigated the role of intellectual humility in a discussion context [15,16]. For example, pairs who discussed a divisive topic changed their attitudes more and perceived their discussion partner more trustworthy, if both were high in intellectual humility [17], and intellectual humility was positively correlated with willingness to learn more about opposing views [18]. Moving beyond past research, the current research examines the discursive qualities of intellectual humility. We sought to determine whether or not intellectual humility can be transcribed and coded from observed body language and discourse. Furthermore, we sought to test whether this measure would reveal increased intellectual humility following completion of an intervention adapted from previous research on reducing psychological defensiveness.

### 1.1. Studying intellectual humility in debate

While there is very extensive linguistic work on the related phenomenon of politeness [19,20], there is, to our knowledge, no linguistic work on humility, arrogance or servility. This gap arises because linguistics focuses on linguistic form, even when drawing on contiguous disciplines such as psychology, sociology, anthropology and philosophy. Politeness phenomena are either systemic to the language (as in honorifics) or are pragmatic choices made by speakers from a finite set of syntactic (e.g. indirect speech acts; [21]) and lexical choices. Arrogance, humility and servility, on the other hand, represent dispositions that have no obvious correlates in speech. Accordingly, we could not build on an existing base of research to code intellectual humility from actual debate.

The humility framework used here was primarily developed bottom-up through close qualitative analysis of our first 20 recorded debates by the linguists on the team. Hundreds of potential types of cues to arrogance and humility were considered before settling on a codable set. These included linguistic cues such as affiliators (e.g. *yeah*, *mmh*), hedges (e.g. *might, I think*) and boosters (e.g. *absolutely*); paralinguistic cues such as pitch, speed, loudness, intonation and laughter; pragmatic cues such as overlapping, yielding turns and interrupting; and non-verbal cues such as head nodding, gesture and gaze. The framework was also informed by two cross-disciplinary workshops held at our higher education institutions (see electronic supplementary material).

The framework considers various ways in which intellectual humility may be manifest in conversation and distinguished from its counterparts, arrogance and servility [22]. Humility occurs when speakers have an opinion and are keen to convey it, but are still open to other views and willing to listen to and engage with them. Arrogance occurs when speakers are sure they are right (irrespective of evidence) and believe that what they have to say is more important than anyone else's opinion. Servility occurs when speakers either have no independent opinion or feel that their opinion is not worth contributing to discussion and will tend to accept what others say.

It is important to recognize that no individual linguistic, paralinguistic, pragmatic or non-verbal feature marks a speaker out as humble, arrogant or servile. The framework developed by our linguistic team [22] suggests that these patterns are manifested along three communicative dimensions: dominance, engagement and conviction. Dominance is the extent to which a speaker seizes and dominates the floor as measured through interruption, yielding turns, and overall floor time. Engagement reflects the extent to which a speaker engages with what others are saying as measured through affiliation and (dis)agreement. Finally, conviction is the extent to which a speaker conveys their opinions as certain, obvious and unchallengeable.

The communicative behavioural dimensions described in the dominance-engagement-conviction (DEC) framework, on their own, do not determine arrogance, humility or servility. For example, while dominance potentially correlates with arrogance, we can distinguish an ‘involved’ speaker who, while dominant in the discussion in terms of floor time and perhaps even interruption, shows a great deal of affiliation to others, which in turn indicates active listening and thus humility. On the other hand, a ‘detached’ speaker, by participating little and interrupting seldom, might show very little dominance, but they might also convey their rare utterances with absolute conviction and show little sign of engagement with what others are saying, both of which suggest arrogance. This complexity requires that we code a series of communicative tendencies observable through discrete discursive phenomena, as described in our Method section and electronic supplementary material, table S1.

### 1.2. Increasing intellectual humility through self-affirmation

With a discursive coding framework in hand, it becomes feasible to examine the psychological underpinnings of intellectual humility in debate. We expected that intellectual humility would be undermined by the psychological defence mechanisms that are activated when the self-concept is challenged. As Rosenberg suggests, ‘people behave in a fashion consistent with the pictures they hold of themselves and interpret any experience contradictory to this self-picture as a threat’ [23, p. 57]. By overtly dismissing the threats and changing the focus of discussion, intellectually arrogant, dogmatic behaviour is one way to prevent threats to self-esteem from dominating consciousness. Similarly, by quickly re-affirming agreement with others without deeper reflection, intellectually servile behaviour diverts attention from these threats to self-supportive social connections.

One way to ameliorate such responses is by enabling people to feel a more coherent sense of self. Intellectually humble people may have a more integrated and accurate view of the self and therefore be less inclined to distort information of themselves to make themselves feel better [24]. One intervention that can improve the subjective sense of self-coherence and integrity is the self-affirmation technique [25]. Cohen & Sherman define self-affirmation as ‘an act that manifests one's adequacy and thus affirms one's sense of global self-integrity’ [26, p. 337]. Self-affirmation theory posits that people are motivated to maintain a global perception of adequacy, rather than perceived worth in specific domains [27].

Self-affirmation can occur in varied ways. The most common intervention involves reflecting on one or two cherished personal values [27]. A brief period of reflection on personal values is presumed to increase the sense of self-integrity, and experimental evidence indicates that this reflection makes people more thoughtful and open-minded in response to persuasive text that challenges their views [28]. Self-affirmation is thought to make available more psychological resources for coping with threat, enabling people to adopt a broader perspective that does not affect self-evaluation. Indeed, self-affirmation reduces defensiveness [29], reduces insecurity [30] and facilitates conflict resolution [31,32]. Overall, then, this work led us to expect that self-affirmation should also increase intellectual humility in face-to-face debate.

### 1.3. The present research

To provide the first direct test of whether or not self-affirmation increases intellectual humility, we asked individuals at an English university and participants who reside closeby to take part in a face-to-face debate. Blind to condition, the experimenter first assigned participants to complete a self-affirmation or control task. Following this manipulation, participants watched a video presenting pre-tested arguments in favour of the current tuition fee structure at English universities and then took part in a video recorded debate about the relative merits of the arguments (including a confederate blind to condition). We then coded the debating behaviour using our new linguistic framework to test whether self-affirmation increased intellectual humility in the debate.

As supplementary tests, participants also completed initial measures assessing diverse psychological individual differences potentially relevant to intellectual humility, including self-report measures of the Big 5 personality traits [33] and of intellectual humility [11].^1^ In addition, we tested whether individuals lower in fragile or defensive self-esteem would exhibit more intellectual humility in the debates, using implicit and explicit measures of self-esteem. Defensive self-esteem occurs when people report high self-esteem, but manifest indications of automatic negative self-evaluations [35,36]. These measures were included also to explore whether or not they moderate or mediate the effects of self-affirmation on intellectual humility in debate (e.g. greater impact among those with more defensive self-esteem; [37,38]). In addition, we measured prosocial emotion, because additional evidence suggests that self-affirmation is more effective when it activates prosocial affect (e.g. empathy, gratitude; [39]). The data and R-code are available at https://osf.io/4qa5w/?view_only=0e9041306e154456af870054e6ee6d58.

## 2. Method

### 2.1. Participants

Although previous research typically found medium to very large effects of value affirmation on mood [39] and persuasion [40], we decided to be conservative and assumed only a medium effect size (Cohen's *d* = 0.50), which resulted in a required sample size of 210 participants for a power of 0.95 and *α* = 0.05. The final sample comprised 303 participants. The mean age of the sample was 23.89 years (s.d. = 10.27, range = 17–71); 200 participants identified as women and 103 as men. Because of a technical issue with the survey program, the personality variables were only obtained from 300 participants.

### 2.2. Materials and procedure

Initial testing took place in computer laboratories. A computer-based survey contained items about demographic information, implicit and explicit measures of self-esteem, and self-report measures of intellectual humility and other traits. Participants then moved to another room and completed the experimental manipulation. Following the manipulation, participants reported their emotions before proceeded to the main observation laboratory to view a short presentation on tuition fees. We chose tuition fees because we assumed that the majority of students would be against it, and it therefore would be a controversial topic. The presentation included eight persuasive arguments in favour of keeping the current tuition fees of £9250 per year. After rating the persuasiveness of each argument, participants took part in a group discussion, which was recorded using audio and video equipment. Participants were then debriefed, thanked and compensated with £12 (€13–14) for their time. Full ethical approval was obtained.

### 2.3. Measures

Internal consistencies (Cronbach's alpha) for all explicit measures consisting of two or more items can be found in table 1.

**Table 1.** Descriptive statistics and correlations across all variable*s. Note.* Cronbach's alphas in main diagonal, if available; IH-Debate: behaviour coded measure of intellectual humility; SI: single-item; Imp: implicit measure; CIHS: comprehensive intellectual humility scale [11]; Gender: 0 = men, 1 = women. The last four variables are coded behaviour from the group discussion and were used to create our behavioural measure of intellectual humility (column/row 1).

variable	*M*	*s.d.*	1	2	3	4	5	6	7	8	9	10	11	12	13	14	15
1. IH-debate	−3.03	8.54															
2. CIHS: independence	3.13	0.91	0.03	0.89													
3. CIHS: revising	4.25	0.41	0	0.19**	0.89												
4. CIHS: respect	4.23	0.42	−0.07	0.27**	0.39**	0.63											
5. CIHS: lack overconf.	3.36	0.49	0.05	0.17**	0.21**	0.23**	0.76										
6. explicit self-esteem	3.54	0.65	0.03	0.13*	0.07	0	−0.17**	0.88									
7. self-esteem: SI Imp	6.62	1.57	0.05	−0.02	0	0.01	−0.09	0.27**									
8. extraversion	3.26	0.85	−0.08	0.03	0.09	0.11	−0.05	0.34**	0.17**	0.80							
9. agreeableness	4.17	0.67	−0.06	−0.12*	0.13*	0.18**	0.23**	0.03	0.03	0.28**	0.79						
10. conscientiousness	3.43	0.81	0.04	0.01	0.06	0.07	0.01	0.21**	0.13*	0.01	−0.05	0.77					
11. neuroticism	2.95	0.80	0.02	−0.25**	−0.09	−0.07	0.02	−0.41**	−0.07	−0.08	0.05	−0.04	0.69				
12. openness	3.85	0.65	−0.07	0.14*	0.17**	0.1	−0.02	0.1	0.1	0.19**	0.06	−0.07	0.02	0.68			
13. political orientation	2.80	1.45	−0.01	0.06	0.02	0.16**	0.04	0	0.07	0	−0.12*	0.09	0.01	−0.06			
14. age	23.9	1.27	−0.04	0.03	−0.12*	−0.17**	−0.16**	0.15*	0.01	−0.02	−0.06	0	−0.08	−0.07	−0.14*		
15. gender	0.66	0.47	0.01	−0.21**	−0.15**	0.02	0.09	−0.04	0.1	0.1	0.28**	−0.04	0.18**	−0.13*	−0.05	−0.02	
16. self-esteem: Imp	1.51	1.25	0.11	−0.09	−0.04	−0.07	−0.05	0.1	0.13*	0.01	0.14*	−0.1	0.11*	0.03	0.04	−0.08	0.19**

#### 2.3.1. Demographic information

Participants indicated their age, gender, political orientation, religion, languages spoken and profession or study subject at university.

#### 2.3.2. Implicit self-esteem

To measure implicit self-esteem, we adapted the Inquisit-by-Millisecond original go/no go task [41], mainly following the design used by Gregg & Sedikides [42]. The procedure is described in detail in the electronic supplementary material.

#### 2.3.3. Self-report intellectual humility

Participants completed the 22-item comprehensive intellectual humility scale [11], which comprises four subscales: independence of intellect and ego (e.g. ‘I feel small when others disagree with me on topics that are close to my heart’ (reversed coded)), openness to revising one's viewpoint (e.g. ‘I am willing to change my opinions on the basis of compelling reason’), respect for others' views (e.g. ‘I am willing to hear others out, even if I disagree with them’) and lack of intellectual overconfidence (e.g. ‘My ideas are usually better than other people's ideas’ (reversed coded)). Responses were given on a 5-point scale ranging from strongly disagree (1) to strongly agree (5).

Explicit self-esteem was measured using Rosenberg's [43] 10-item self-esteem scale. Examples of items are ‘I feel that I am a person of worth’, and ‘All in all, I am inclined to feel that I am a failure’ (reversed coded). Responses were given on a 5-point scale ranging from strongly disagree (1) to strongly agree (5).

Personality traits were assessed with the 20-item mini-international personality item pool [33], which presents four items for each of the Big 5 dimensions of personality: extraversion, agreeableness, conscientiousness, neuroticism and intellect/imagination. Responses were given on a 5-point scale ranging from strongly disagree (1) to strongly agree (5).

Immediately after the experimental manipulation, participants completed a measure originally administered by Crocker *et al*. [39] to assess a range of emotions they may have experienced during the writing task. The emotions were love, joyful, giving, empathic, angry, sympathy, grateful, proud, sad, clear, vulnerable, critical, humble, selfish, scared, content, confused and connected. Responses were given on a 5-point scale ranging from very slightly (1) to extremely (5).

#### 2.3.4. Single-item implicit measure of self-esteem

We presented a previously validated single-item implicit measure of global self-esteem, which correlates with other implicit measures of self-esteem [44]. The question asked simply, ‘how much do you like your name?’ Responses were given on a 9-point scale, ranging from 1 (dislike extremely) to 9 (like extremely).

### 2.4. Experimental manipulation

Blind to condition, the experimenter randomly assigned the participants to either the self-affirmation or control condition after they had completed the computer-based tasks. Participants within a debate group were assigned to the same condition. In the self-affirmation condition, participants were first given a list with 19 value types [45] and ranked the five values they found most important. Next, participants were instructed to ‘write for a few minutes in the box about the value you ranked as number 1 – the most important value to you, explain why it is so important to you and how this value guides your behaviour’. In the control condition, participants wrote why they like or dislike five beverages (e.g. tea, milk).

### 2.5. Group discussion

#### 2.5.1. Persuasive arguments

We formulated a list of eight statements that supported the retention of the existing UK undergraduate tuition fees of £9250. Arguments for retention of the fees were informed by opinions voiced on a number of student social media platforms and fora. A list of 15 arguments were evaluated in a pilot study using the crowdsourcing platform Prolific Academic (prolific.co). Participants (*n* = 50) were asked to rate the relative strength of this set of statements supporting undergraduate tuition fees. To ensure there would be content to debate, the final set consisted of the four weakest and four strongest arguments as rated by the respondents (see electronic supplementary material, Information). These arguments were included together in a Powerpoint slide presentations. While watching the presentation, participants completed a response booklet containing rating scales assessing the persuasiveness of each of the arguments [40] on a scale from 1 (not at all persuasive) to 7 (very persuasive). Participants then indicated in the response booklet whether they are in favour, against or undecided regarding the UK tuition fee model of £9250 (51.5% were against tuition fees, 25.8% undecided and 22.7% in favour).

#### 2.5.2. Recorded 15-minute debate

A 15-minute recorded debate took place following the slide presentation. The experimenter asked the participants to agree on the strongest and weakest arguments. The experimenter stated that there were no right or wrong answers and that the discussion would be terminated after 15 minutes, regardless of whether or not they had managed to reach a consensus. A confederate in the group, who was also blind to condition, attempted to resist the general direction the group was taking, not agreeing with the group decisions to ensure that the group did not reach consensus on the controversial issue without dialogue. Despite the instruction to agree on the strongest and weakest arguments, most groups deviated at some point during the debate and ended up discussing tuition fees in general. The experimenter or confederate did not intervene because a less structured discussion allowed participants to act more in line with their personality (cf. [46]).

To make the participants believe that the confederate was also a participant, they were told at the beginning of the study that one participant was running late and would join when they could. Typically, the confederate arrived in time for the writing task (i.e. experimental manipulation). The confederate always received the control writing task to make them blind to the condition of the real participants. The group sizes varied between four (75 groups), three (39 groups) and two (three groups) participants, including one confederate. Each participant wore an individual microphone for optimal audio recording, two camcorders recorded HD video footage of the interactions, and two back-up cameras recorded the discussion from the room corners.

### 2.6. Coding of group discussion

Three linguistics experts, who were blind to the condition, coded the group discussion behaviour separately for all participants (including the confederate). The exact procedure is outlined in the electronic supplementary material and in [22]. Relevant for the present research are the five categories described in detail in our electronic supplemental material: affiliate, affiliate agree, affiliate disagree, agreement and boosted conviction. Because coding the responses is very time consuming, only a subset of our 81 participants was coded by two additional raters, who were blind to the responses of the first raters and the experimental condition. This procedure follows other research in which complex coding was performed (e.g. [47]). As a measure of inter-rater reliability, we computed intra-class coefficients (ICC), which were high for five categories we examined prior to derivation of our intellectual humility index: affiliate (ICC = 0.994, 95% CI [0.991, 0.996]), affiliate agree (ICC = 0.98, 95% CI [0.97, 0.99]), affiliate disagree (ICC = 0.88, 95% CI [0.82, 0.92]), agreement (ICC = 0.95, 95% CI [0.92, 0.97]) and boosted conviction (ICC = 0.96, 95% CI [0.94, 0.97]). For all analyses, we used the coding from the first set of raters for consistency; the results were almost identical when we replaced the coding from the first set of raters with those from the second set (e.g. table 1).

Prior to our analysis, the researchers met to arrive at an approach for calculating intellectual humility from the coding. As described in the electronic supplementary material, we first developed the theoretical framework and then revised it after discovering coding difficulties for the dominance dimension of our framework. We agreed that intellectual humility (IH) was evident in *higher* qualified engagement (e.g. ‘yeah that's right’, ‘okay but’), *lower* boosted conviction (e.g. less of ‘always’, ‘obviously’), and *lower* servility (e.g. less of primarily ‘yes’ and ‘yeah’, and other unqualified affirmations):

IH=Qualified Engagement−Boosted Conviction−Servility.

In terms of our coding, this approach entailed calculating the amount of qualified engagement as a sum of affiliate-agree, affiliate-disagree and agreement, the amount of boosted conviction, and the amount of servility as the *proportion* of unqualified affirmation (i.e. affiliate) versus the total qualified engagement and boosted conviction. This approach yielded the following formula, which was then applied to the data:

$$\begin{array}{rl} \mathrm{IH}= & \left[\mathrm{Affiliate}-\mathrm{agree}+\mathrm{Affiliate}-\mathrm{disagree}+\mathrm{Agreement}]\text{–}[\mathrm{Boosted}\;\mathrm{Conviction}\right]\\ & \text{–}[\left(\mathrm{Affiliate})/(1+\mathrm{Boosted}\;\mathrm{Conviction}+\mathrm{Affiliate}\;\mathrm{agree}+\mathrm{Affiliate}\;\mathrm{disagree}+\mathrm{Agreement}\right)]. \end{array}$$

## 3. Results

Data were analysed using SPSS and R, with the support of the packages lme4 (v. 1.1–27.1; [48]), ggstatsplot (v. 0.8.0; [49]) and psych (v. 2.1.6; [50]).

### 3.1. Correlational analyses

Table 1 displays the descriptive statistics, internal consistencies (if applicable), and correlations for most measures.

#### 3.1.1. Comprehensive intellectual humility scale

As expected, there were positive correlations between the four intellectual humility subscales (*r*s ≥ 0.17–0.39). As shown in table 1, these scales and the calculation of intellectual humility from the dialogue were uncorrelated.

#### 3.1.2. Self-esteem

Table 1 shows that participants who exhibited higher scores on the explicit measure of self-esteem also scored higher on the name-liking indirect measure of self-esteem and on conscientiousness while scoring lower on neuroticism and on the lack of overconfidence subscale in the comprehensive intellectual humility scale. Consistent with past research [44], the name-liking single-item implicit measure of self-esteem exhibited small positive correlations with the implicit measure of self-esteem.

#### 3.1.3. Personality traits (Big 5)

As shown in table 1, there were significant correlations between the four dimensions of the Comprehensive Intellectual Humility Scale and three of the personality traits: agreeableness, openness and neuroticism. For example, higher agreeableness was associated with more revising, respect and lack of overconfidence, but negatively with independence. The Big 5 were mostly uncorrelated with the implicit measures and our debate-based measure of intellectual humility.

#### 3.1.4. Demographic variables

Table 1 shows that individuals on the political right-wing scored more highly on the respect dimension of the intellectual humility measure than individuals on the political left. Older participants and men scored lower on the revision dimension of intellectual humility, with older participants also scoring lower on the respect and lack of overconfidence dimensions. Age, gender and political views were not reliably associated with the coding of intellectual humility from the participant discussions.

### 3.2. Effects of self-affirmation

Table 2 displays the results of the self-affirmation versus control comparisons for all relevant dependent variables.

**Table 2.** Between-subject comparisons between control and affirmation condition. *Note*. s.d.: standard deviation, U3: Cohen's U3 (percentage of people in affirmation group who score higher than the average of the control group). All reported *p*-values are two-tailed. Bold indicates statistical significance at the *p* < 0.05 level.

	control		affirmation		*t*	*p*	*d*	*U3*
	*M*	*s.d.*	*M*	*s.d.*				
**IH-debate**	**−3.82**	**9.14**	**−1.61**	**7.29**	**2.24**	**0.0258**	**0.27**	**60.56**
love	2.24	1.26	2.36	1.28	0.82	0.4154	0.09	53.78
**joyful**	2.78	1.12	2.36	1.17	−3.10	0.0021	−0.36	35.93
**giving**	2.15	1.22	2.75	1.19	4.32	0.0000	0.50	69.22
**empathic**	2.08	1.19	3.06	1.28	6.86	0.0000	0.80	78.73
angry	1.26	0.65	1.39	0.79	1.50	0.1353	0.17	56.89
**sympathy**	1.71	1.06	2.56	1.34	6.09	0.0000	0.71	75.98
**grateful**	2.57	1.33	3.07	1.39	3.14	0.0019	0.37	64.28
**proud**	2.07	1.18	2.62	1.32	3.80	0.0002	0.44	67.02
**sad**	1.34	0.75	1.81	1.07	4.43	0.0000	0.51	69.53
clear	3.30	1.13	3.07	1.18	−1.77	0.0773	−0.21	41.84
**vulnerable**	1.38	0.78	1.83	1.08	4.08	0.0001	0.47	68.13
critical	2.48	1.30	2.38	1.25	−0.66	0.5076	−0.08	46.92
**humble**	1.94	1.05	2.43	1.13	3.90	0.0001	0.45	67.44
**selfish**	1.27	0.66	1.54	0.85	3.06	0.0024	0.35	63.83
**scared**	1.11	0.32	1.38	0.81	3.77	0.0002	0.43	66.69
**content**	3.14	1.06	2.72	1.16	−3.33	0.0010	−0.39	34.99
confused	1.86	0.98	1.66	0.97	−1.78	0.0762	−0.21	41.82
**connected**	2.16	1.19	2.58	1.12	3.11	0.0020	0.36	64.12

#### 3.2.1. Intellectual humility in the debates

**Figure 1.**
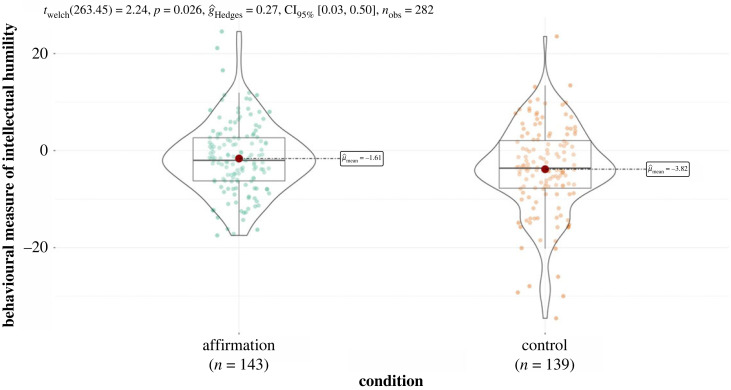
Effect of self-affirmation on the behavioural measure of intellectual humility.

As predicted, self-affirmation increased behavioural intellectual humility, *t*_280_ = 2.25, *p* = 0.013, *d* = 0.27, Cohen's U3 = 60.56, indicating that 60.56% of participants in the affirmation condition exhibited more intellectual humility in the debates than the average of the control condition (figure 1). The analysis had 80% power to detect an effect size of *d* = 0.33 (with *α* = 0.05, two-tailed). This finding remained robust when we conducted a linear–mixed effect model that took the nested structure of the data into account (participants nested in groups nested with different confederates): effect of condition with participants nested in groups, *B* = 2.38, s.e. = 1.19, *p* = 0.048, and effect of condition with participants nested in groups, which are nested with a confederate, *B* = 2.52, s.e. = 1.16, *p* = 0.039. Both models had random intercepts and slopes.

#### 3.2.2. Emotion

Consistent with Crocker *et al*.'s [39] findings, there was evidence that self-affirmation increased prosocial emotions (table 2). Participants who completed the self-affirmation task reported more prosocial emotion; they reported feeling more giving, empathy, sympathy, gratitude, humble and connected, although they did not report more love. Surprisingly, however, participants who completed the self-affirmation task also reported feeling more pride, vulnerable, sad, selfish, scared and less content. Further tests, for example including only prosocial emotions, supported the robustness of these findings (see electronic supplementary material).

#### 3.2.3. Argument persuasiveness

To test whether there are differences between the affirmed and control condition in how persuasive they found the arguments, we performed a 2 (affirmed versus control) × 3 (against versus in favour versus undecided) × 2 (weak versus strong arguments) mixed ANOVA. The last factor was within-subjects. There was a main effect of argument strength, *t*_281_ = 4.57, *p* < 0.001, such that the strong arguments were judged as more persuasive (*M* = 3.78, s.d. = 1.03) than the weak arguments (*M* = 3.51, s.d. = 1.02). Nonetheless, the difference between strong and weak arguments was small, *d*z = 0.26. No other effects or interactions were significant, *p*s > 0.36. We found the same pattern of results if we only used the two strongest and two weakest arguments from the present sample.

#### 3.2.4. Defensive self-esteem

To test whether people with a defensive self-esteem (low implicit but high explicit self-esteem) reported less intellectual humility and manifested less intellectual humility in the group discussion, we performed a series of moderated regression analyses, following Haddock & Gebauer [35]. Specifically, we entered the linear terms of explicit and implicit self-esteem as well as their interaction as predictors in multiple regression analyses with the four dimensions of intellectual humility and the behavioural coding of intellectual humility as dependent variables. The interaction term did not reach significance in these analyses, also when we used our single item implicit measure of implicit self-esteem.

### 3.3. Mediational analysis

We tested whether the effect of self-affirmation on the behavioural measure of intellectual humility was dependent on the effect on prosocial emotion. We conducted a mediation analysis with condition as predictor, the aggregate index of prosocial emotion as the mediator, and the measure of intellectual humility in debate as the outcome. The bootstrapped (*n* = 5000) indirect effect was not significant, 95% CI [−0.92, 0.49]. In addition, all 18 emotion items and the prosocial emotions combined were uncorrelated with the behavioural measure of intellectual humility, *r*s ≤ 0.10. Thus, we obtained no evidence that prosocial emotion mediates the impact of the self-affirmation intervention on intellectual humility in debate.

## 4. Discussion

Intellectual humility is an important virtue in face-to-face debate, but the development of interventions to boost intellectual humility in this context has been impeded by the lack of a framework for conceptualizing and measuring intellectual humility in dialogue. Building on a collection of insights for conceptualizing intellectual humility (with an emphasis on humility versus arrogance and servility), we coded intellectual humility from dialogue in face-to-face debates on a contentious issue and tested whether an intervention previously used to decrease psychological defensiveness increased intellectual humility in this context. Results indicated that the intervention, which affirmed the self-concept by inducing participants to think about their most important values, subsequently increased intellectual humility. Consistent with Crocker *et al*. [39], this self-affirmation intervention also increased prosocial emotions.

Other aspects of the findings were surprising. First, the effect of self-affirmation on intellectual humility was not mediated by its impact on prosocial emotion—these effects were independent. This lack of mediation might be consistent with the larger pattern of effects on emotions reported by participants. In addition to feeling more prosocial emotion after self-affirmation, they also reported feeling more pride, vulnerable, sad, selfish, scared and less content. These latter findings may reflect a more complex effect of self-affirmation on emotions, perhaps as a result of social interconnectedness activated in the task, such as pride in one's connections alongside the recognition of self–other conflicts in this interconnectedness (e.g. selfish and vulnerable).

Alternatively, prosocial emotion might simply fail to account for effects of self-affirmation in the debating context, in line with previous research that also did not find a mediating effect of mood [32]. Another process may be crucial: it has been suggested that there is a broad impact of self-affirmation on the psychological resources available for coping with threat, enabling people to adopt a broader perspective that does not affect self-evaluation [51]. Like past research (e.g. [40]), we focused on creating a context that elicited disagreement, but this did not entail setting out to create a sense of threat *per se*. It might be the case that a more focused examination of felt threat and anxiety in future research would reveal support for the hypothesis that self-affirmation builds resources that attenuate the impact of these emotions. The literature on self-affirmation effects also points to an increased sense of self-integrity [25,28,52]. In theory, self-affirmation increases people's subjective sense of self-integrity, which frees them to be less wary of threats to their self-views and more open-minded. We would like to have also tested this explanation in our design, but we had not seen any operationalization of self-integrity that was both widely validated and portable to our research design. In addition, we had not included a self-report measure of self-affirmation inclination [53], which might itself have provided correlational evidence for associations with self-report intellectual humility and intellectual humility coded from our debates.

Two other findings are noteworthy. First, the self-report measurement of explicit intellectual humility did not predict intellectual humility as coded from our debates. This finding aligns with various researchers' concerns about self-report measures of intellectual humility (e.g. [7,24]), with meta-analytic research that found only very small or non-significant associations between personality and specific behaviour (e.g. [54]), and with recent evidence that self–other agreement for intellectual humility is very low even among students who worked together on projects [55,56]. Our findings also align with the expectations of the linguists in our research team. From the linguistic perspective, people *perform* in speech: they deliberately try to convey a certain persona. For example, if they ‘know’ they are open to others' views, they may see little need to perform being humble. From this vantage point, speech is never the transparent manifestation of mind, and of course there is the problem of self-deception in the self-report measurements themselves. Nevertheless, we expect that, when measured as an individual difference variable, intellectual humility is likely to reflect some contexts more than others. That is, the self-reports may be based on social engagements that are accessible and available to participants. A formal social debating context, as used in our design, is unlikely to be one of those contexts that spring to mind. Furthermore, trait measures of intellectual humility might also predict multiple aggregated instances of relevant behaviour, but our study was not intended to examine intellectual humility across diverse situations. In addition, we had not included a self-report measure of self-affirmation inclination [53], which might itself have provided correlational evidence for associations with self-report intellectual humility and intellectual humility coded from our debates.

The second finding of note is that it is interesting that self-affirmation did not cause *differential* responding to the strong and weak arguments included in the debates. In other words, it did not cause consistently more agreement with the stronger arguments and consistently less agreement with the weaker arguments. If this effect had occurred, it would have extended prior evidence of greater open-minded scrutiny of arguments presented in a videotape containing strong and weak arguments following self-affirmation [40]. Although this effect was obtained in only a subsample of Correll *et al*.'s participants (those who considered the target issue to be highly important to them), we recognized this possible impact of self-affirmation beforehand and therefore tested it. However, we regarded it as a very weak possibility because of a crucial difference between the past research and the design of our experiment. Specifically, Correll *et al*.'s participants did not discuss the arguments directly in their own debate. Our introduction of this social dialogue about the arguments forced high scrutiny of the arguments for participants in both experimental conditions, and this message processing constraint has been shown to eliminate argument strength effects [57]. Consequently, there was little message processing latitude for self-affirmation to elicit more differentiation between strong and weak arguments in this context.

Of course, self-affirmation is only one potential intervention to increase intellectual humility in debate, and it will take many more investigations to further understand ways to increase intellectual humility in debate and to predict its occurrence. We are nonetheless intrigued by the potential to adapt our in-person methodology to an online context. The coding scheme used here can easily be adapted to code text in online forum discussions and other contexts where acrimony might arise. Furthermore, it would be useful to see whether similar effects of self-affirmation occur for diverse topics, such as ones that are more reflective of ideological, national, ethnic or other group identities [58–60]. While tuition fees were important for our sample (mostly students), they were posed as a future problem and students had already agreed to them while signing up to their degree. It would be interesting to test the effect of self-affirmation on a more heated topic of high personal relevance, and with people who are more controversial than our mildly provocative confederate. In general, self-affirmation makes people relatively more open to information and ideas that would otherwise prove threatening to their identity [52], and online dialogue is an important context in which to examine this process. Such a study would further address calls for interventions to engage more with or be more receptive towards opposing views [61]. Another intriguing possibility is to include value affirmation at public engagement activities, perhaps using an online measure of values like one we used at the Royal Society 2022 Science Exhibition (http://discovervalues.org/). Such public engagement events are great places to run scientific studies (e.g. [62]). They enable tests of whether findings obtained in the laboratory or online generalize to field contexts.

In sum, our research demonstrates that intellectual humility in a debate can be enhanced through a relatively simple intervention. Put numerically, 60.6% of participants in the affirmation condition showed more intellectual humility in debate than the average person in the control condition. This finding, and to some extent the concomitant effect on prosocial emotion, reveal promising implications for improving the quality of dialogue on controversial issues. We hope future research follows up on these implications. Although the study of intellectual humility is difficult due to complexities in its conceptualization and measurement, any progress in understanding its antecedents has important potential to aid democratic processes in addressing the pivotal issues of our time.

## Data Availability

The data and R-code to reproduce the analyses are available at https://osf.io/4qa5w/?view_only=0e9041306e154456af870054e6ee6d58. The data are provided in electronic supplementary material [63].

## Footnotes

1 To complement the self-report measures, we also attempted to pilot an adaptation of the affect misattribution paradigm (AMP; [34]) to implicitly assess intellectual humility and intellectual arrogance but obtained no reliable evidence from this pilot and therefore only report it in the electronic supplementary material.
